# Genome-Wide Identification Reveals That *Nicotiana benthamiana* Hypersensitive Response (HR)-Like Lesion Inducing Protein 4 (NbHRLI4) Mediates Cell Death and Salicylic Acid-Dependent Defense Responses to Turnip Mosaic Virus

**DOI:** 10.3389/fpls.2021.627315

**Published:** 2021-05-25

**Authors:** Xinyang Wu, Yuchao Lai, Shaofei Rao, Lanqing Lv, Mengfei Ji, Kelei Han, Jiajia Weng, Yuwen Lu, Jiejun Peng, Lin Lin, Guanwei Wu, Jianping Chen, Fei Yan, Hongying Zheng

**Affiliations:** ^1^State Key Laboratory for Managing Biotic and Chemical Threats to the Quality and Safety of Agro-Products, Key Laboratory of Biotechnology in Plant Protection of Ministry of Agriculture and Zhejiang Province, Institute of Plant Virology, Ningbo University, Ningbo, China; ^2^College of Agriculture and Biotechnology, Zhejiang University, Hangzhou, China

**Keywords:** HR-like lesion inducing protein, cell death, salicylic acid, turnip mosaic virus (TuMV), genome-wide identification

## Abstract

Hypersensitive response (HR)-like cell death is an important mechanism that mediates the plant response to pathogens. In our previous study, we reported that NbHIR3s regulate HR-like cell death and basal immunity. However, the host genes involved in HR have rarely been studied. Here, we used transcriptome sequencing to identify *Niben101Scf02063g02012.1*, an HR-like lesion inducing protein (HRLI) in *Nicotiana benthamiana* that was significantly reduced by turnip mosaic virus (TuMV). HRLIs are uncharacterized proteins which may regulate the HR process. We identified all six *HRLIs* in *N. benthamiana* and functionally analyzed *Niben101Scf02063g02012.1*, named *NbHRLI4*, in response to TuMV. Silencing of *NbHRLI4* increased TuMV accumulation, while overexpression of NbHRLI4 conferred resistance to TuMV. Transient overexpression of NbHRLI4 caused cell death with an increase in the expression of salicylic acid (SA) pathway genes but led to less cell death level and weaker immunity in plants expressing *NahG*. Thus, we have characterized NbHRLI4 as an inducer of cell death and an antiviral regulator of TuMV infection in a SA-mediated manner.

## Introduction

In response to infection, plants rapidly activate HR-like cell death at the primary infection site, which helps restrict the movement of various pathogens (Heath, 2000; Greenberg and Yao, 2004). HR-like cell death is usually accompanied by the activation of other defense reactions, including the accumulation of SA, jasmonic acid (JA), and ethylene (ETH) and opening of ion channels and thus comprehensively regulates plant resistance (Beers and McDowell, 2001; Lam et al., 2001; Vlot et al., 2009).

The SA pathway is one of those deeply involved in HR-like cell death to regulate plant resistance to pathogens, and several host factors have recently been shown to be involved in this SA-mediated HR response. In soybean (*Glycine max*), silencing *GmMEKK1* lead to strong HR-like cell death, with the accumulation of SA, H_2_O_2_, and defense-related genes (Xu et al., 2018). Some transcriptional factors (TFs) were also reported to participate in this process. In *Arabidopsis*, AtMYB30 is involved in a signaling cascade process that regulates SA synthesis and further modulates cell death (Raffaele et al., 2006). Overexpression of BrERF11 transcription factor (TF) in Chinese cabbage (*Brassica rapa* L.) conferred resistance to the bacterium *Ralstonia solanacearum* coupled with HR, and upregulation of defense-related genes including HR marker genes and both SA- and JA-dependent pathogen-related genes (Lai et al., 2013).

Several host genes involved in the HR process against plant viruses have also been characterized. In potato, the *Nb* gene triggers local and systemic defense responses including HR in response to PVX (Sánchez et al., 2010). After tospovirus infection of tomato (*Solanum lycopersicum*), Sw-5b induces HR by recognizing the viral movement protein NSm (Zhu et al., 2017).

Turnip mosaic virus is a positive single-stranded RNA virus in the family *Potyviridae* which encodes at least 11 different mature proteins. TuMV causes serious harm to a broad range of plants and is a major threat to the vegetable industry worldwide (Movahed et al., 2017; Wu et al., 2018). TuMV inoculation of *N. benthamiana* results in local necrotic spots and systemic necrosis, and it has been shown that heterotrimeric G-proteins promote this host cell death as a defense response to TuMV (Brenya et al., 2016). We have also previously reported that NbHIR3s induces cell death *via* an SA-dependent pathway and are essential for the resistance this provides (Li S. et al., 2019).

Despite these findings, the host factors involved in the cell death response to TuMV are still largely unknown. HRLI is an uncharacterized protein, but in soybean, GmHRLI1 interacts with SMV P3 protein. However, the function of HRLIs in virus infection is largely unknown. In this study, we used transcriptome sequencing to detect *HRLI* and identified six *HRLIs* in a genome-wide search of *N. benthamiana*. We found that *NbHRLI4* caused cell death and negatively regulated the infection of TuMV, but in plants expressing *NahG*, levels of cell death and defense were both weakened, suggesting that the SA pathway is vital for *NbHRLI4*-mediated immunity. The results will help better understand the function of *HRLIs* in response to TuMV.

## Materials and Methods

### Plant Materials, Virus Inoculation, and SA Treatment

Wild-type (WT) and *NahG* transgenic *N. benthamiana* plants (donated by Dr. Yule Liu, Tsinghua University, China) were grown in a greenhouse under a 16-h light/8-h dark regime at 25 ± 2°C.

*Nicotiana benthamiana* leaves were mechanically inoculated with TuMV or infiltrated with *Agrobacterium tumefaciens* carrying the TuMV-GFP vector as described (Wang et al., 2019). Plants were examined daily for virus symptoms and GFP fluorescence under UV light. SA (Sigma-Aldrich Code No. 247588; 10 μM in 0.1% (*v*/*v*) ethanol) was sprayed to both the abaxial and adaxial sides of leaves 48 h before inoculation with TuMV; 0.1% ethanol was used as the negative control.

### Transcriptome Sequence

The transcriptome sequence was performed in LC-Bio (Hangzhou, China). Four leaf age of *N. benthamiana* were infiltrated with TuMV-GFP infectious clone, and 6 days postinfiltration, the systemic leaves were used for transcriptome sequence. Total RNA was extracted using Trizol reagent (Invitrogen, CA, United States) following the manufacturer’s procedure. Approximately 10 μg of total RNA representing a specific adipose type was subjected to isolate poly(A) mRNA with poly-T oligo-attached magnetic beads (Invitrogen). Following purification, the poly(A)- or poly(A)+ RNA fractions are fragmented into small pieces using divalent cations under elevated temperature. The cleaved RNA fragments were then reverse-transcribed to create the final cDNA library in accordance with the protocol for the mRNA-Seq sample preparation kit (Illumina, San Diego, CA, United States); the average insert size for the paired-end libraries was 300 bp (±50 bp). We then performed the paired-end sequencing on an Illumina Novaseq^TM^ 6000 at the LC-Bio (Hangzhou, China) following the vendor’s recommended protocol. RNA libraries were prepared for sequencing using standard Illumina protocols. Additional information is listed in Supplementary Table 1. The data were uploaded in NCBI-GEO (Accession No. GSE167415).

### Genome-Wide Identification and Bioinformatics Analysis

The genome-wide identification was conducted by two round Blast against AtHRLI protein sequences with the *N. benthamiana* genome v1.0.1 (Bombarely et al., 2012) by TBtools as previously described (Chen C. et al., 2020; Wu et al., 2020). We constructed a neighbor-joining (NJ) phylogenetic tree of NbHRLIs with 1,000 bootstrap replicates using MEGA7.0. Gene structure and domains were analyzed using TBtools (Chen C. et al., 2020) and NCBI-Batch CDD (Marchler-Bauer et al., 2015, 2017). Motif composition was predicted by MEME (Bailey et al., 2009), and multiple sequence alignment was done using DNAMAN. *Cis*-acting elements and potential TFs were predicted by PlantCARE (Lescot et al., 2002) and PlantRegMap (Jin et al., 2017), respectively.

### Vector Construction and *Agrobacterium* Infiltration

The TRV-based VIGS system was used to silence *NbHRLI4* (Liu et al., 2002). An ∼300-bp fragment of *NbHRLI4* was cloned into TRV-RNA2 to generate TRV-NbHRLI4, and the empty vector TRV-00 was used as a negative control. At 10–14 days postinoculation (dpi), plants infected with TRV-NbHRLI4 or TRV-00 were used for further experiments. For overexpression of genes, the entire CDS was cloned, fused with tags (3 × Flag, GFP) and then introduced into the PCV vector as previously described (Wang et al., 2019; Han et al., 2020). Vectors were transformed into *A. tumefaciens* GV3101 and infiltrated into *N. benthamiana* leaves for transient overexpression. At 60–72 h postinfiltration (hpi), the leaves were collected for western blotting (OD_600_ = 0.5) or confocal observation (OD_600_ = 0.1). All primers used in this study are listed in Supplementary Table 1.

### Total RNA Extraction and Quantitative Real-Time PCR

Total RNAs were extracted with Trizol (Invitrogen, United States) according to the manufacturer’s instructions. First-strand cDNA was synthesized from 0.5 mg of RNA with the PrimeScript RT reagent kit (TaKaRa). Three independent biological replicates and three technical replicates were used for real-time PCR (RT-qPCR), with *N. benthamiana* Ubiquitin C (UBC) (AB026056.1) as the internal reference gene. A Roche LightCycler^®^480 Real-Time PCR System with SYBR-green fluorescence was used for the reaction, and the results were analyzed by the ΔΔCT method. All primers used for RT-qPCR are listed in Supplementary Table 1. The mean expression values were calculated from three independent biological replicates and analyzed using *t*-test (two samples) or *F*-test (multiple samples).

### Western Blotting

A mixture of total proteins from at least three different samples was extracted with lysis buffer (100 mM Tris–HCl pH 8.8, 60% SDS, 2% β-mercaptoethanol). Briefly, 40 mg plant samples were lysed in 100 μl lysis buffer and placed on ice for 30 min. Protein samples were then centrifuged at 13,000 rpm for 15 min at 4°C, and then the supernatant was removed by aspiration and boiled. Seven microliters of protein was separated on 12% SDS-PAGE gels for detection with primary antibodies (anti-flag (0912-1, Huabio, China), anti-GFP (ET1607-31, Huabio, China), or anti-TuMV-CP (1075-06, Adgen, United Kingdom) and secondary antibodies (antimouse or antirabbit) (Sigma-Aldrich, St. Louis, MO, United States). Dilution rates were 1:2,500 for primary antibodies and 1:10,000 for secondary antibodies. After incubation with a secondary antibody, proteins were visualized with NBT/BCIP buffer (Sigma) at room temperature. The loading control was visualized by the band intensity of the internal reference protein Rubisco stained with a fuchsia dye. The relative amount of accumulated protein was calculated by comparing the protein band intensity with the loading control using Image J.

### Coimmunoprecipitation

Coimmunoprecipitation (Co-IP) assays were conducted as previously described (Yang X. et al., 2019; Chen B. et al., 2020). PCV-NbHRLI4-flag was coexpressed with PCV-TuMV-P3-GFP, PCV-TuMV-P3C-GFP, PCV-TuMV-P3N-GFP, PCV-TuMV-P3N-PIPO-GFP, or PCV-GUS-GFP in equal volumes (OD_600_ = 1.0) and infiltrated into leaves. One hundred fifty milligrams of leaf tissues was lysed in 600 μl lysis buffer (10% glycerol; 25 mM Tris-HCl (pH 7.5); 1 mM EDTA; 150 mM NaCl; 10 mM DTT; 1 mM PMSF; 0.1% Nonident P40; protease inhibitor cocktail) and placed on ice for 30 min. After protein samples were centrifuged at 13,000 rpm for 15 min at 4°C, the supernatant was removed by aspiration and incubated with GFP-Trap^®^_MA beads (Chromotek) according to the manufacturer’s instructions. After incubation, the beads were washed five times with lysis buffer and immunoblotted with GFP or flag antibodies.

### Yeast Two-Hybrid Assays

Yeast two-hybrid (Y2H) analysis was performed following the Clontech yeast protocol handbook. The yeast NMY51 competent cells were prepared using the lithium acetate method (Song et al., 2016). The yeast expression vectors pBT-NbHRLI4, pPR3-TuMV-P3, pPR3-TuMV-P3C, pPR3-TuMV-P3N, pPR3-TuMV-P3N-PIPO, and pPR3-N were constructed and cotransformed into yeast cells. The yeast cells were cultured on a selective medium lacking tryptophan and leucine (SD/-Trp-Leu) to confirm the correct cotransformation. The transformed yeast cells were then cultured on deficient medium (SD/-Ade-His-Leu-Trp) to test the interactions of the expressed proteins.

### Confocal Microscopy and Bimolecular Fluorescence Complementation

The plant tissues expressing proteins were imaged using Leica TCS SP5 confocal microscope (Leica Microsystems, Bannockburn, IL, United States).

The BiFC assays were carried out as previously described (Yang X. et al., 2019; Jiang et al., 2020). NbHRLI4 and its mutants were fused with an N-terminal fragment of YFP, while TuMV-P3, TuMV-P3C, TuMV-P3N, and TuMV-P3N-PIPO were fused with a C-terminal fragment of YFP. The two agrobacterial cultures were mixed equally to OD_600_ = 0.1 and infiltrated into *N. benthamiana* leaves. At 60–72 hpi, the leaf tissues were observed under a confocal microscope. Target proteins combined with GUS were used as negative controls. YFP excitation was produced using a 514-nm laser with 3% power.

### Trypan Blue Staining, H_2_O_2_ Detection, and Electrolyte Leakage Assays

Leaves were submerged in trypan blue staining solution (6 vol. of ethanol, 1 vol. of water, 1 vol. of phenol, 1 vol. of glycerol, 1 vol. of lactic acid, 0.067% (*w*/*v*) trypan blue) and heated in a boiling water bath for 2–5 min. The solution was replaced with chloral hydrate after cooling, and the samples were shaken until fully destained. 3,3′-Diaminobenzidine (DAB)-HCl (Sigma-Aldrich) was used to detect H_2_O_2_ visually in leaves as previously described (Daudi and O’Brien, 2012; Yang et al., 2020).

The electrolyte leakage assays were conducted as previously described (Aguilar et al., 2019). In brief, 24 leaf disks (diameter 0.3 cm) were excised, rinsed briefly with water and then floated on 5 ml ddH_2_O for 5–6 h at room temperature. The water conductivity resulting from electrolyte leakage (reading 1) was then measured with a conductivity meter (INESA, Shanghai, China). After boiling for more than 20 min and natural cooling, the water conductivity resulting from the total ions was measured again (reading 2). Electrolyte leakage was calculated as [(reading 1)/(reading 2) × 100].

## Results

### Genome-Wide Identification of NbHRLI Family Members

In order to identify cell death-related genes involved in TuMV infection, the transcriptome sequence of *N. benthamiana* plants infected with TuMV was compared with that of mock-inoculated plants. A total of 514,054,210 reads were obtained, and differentially expressed genes (DEGs) were selected using the criteria | log2 fold change| ≥ 1 and *P* < 0.05; 2,905 upregulated and 3,224 downregulated DEGs were identified of which 19 were cell-death related based on their annotation (Supplementary Table 2). *Niben101Scf02063g02012.1*, annotated as an HRLI, was found to be significantly downregulated by TuMV. HRLIs may be associated with the HR pathway, but identification of their family members and their molecular function in plant immunity have rarely been studied.

**FIGURE 1:**
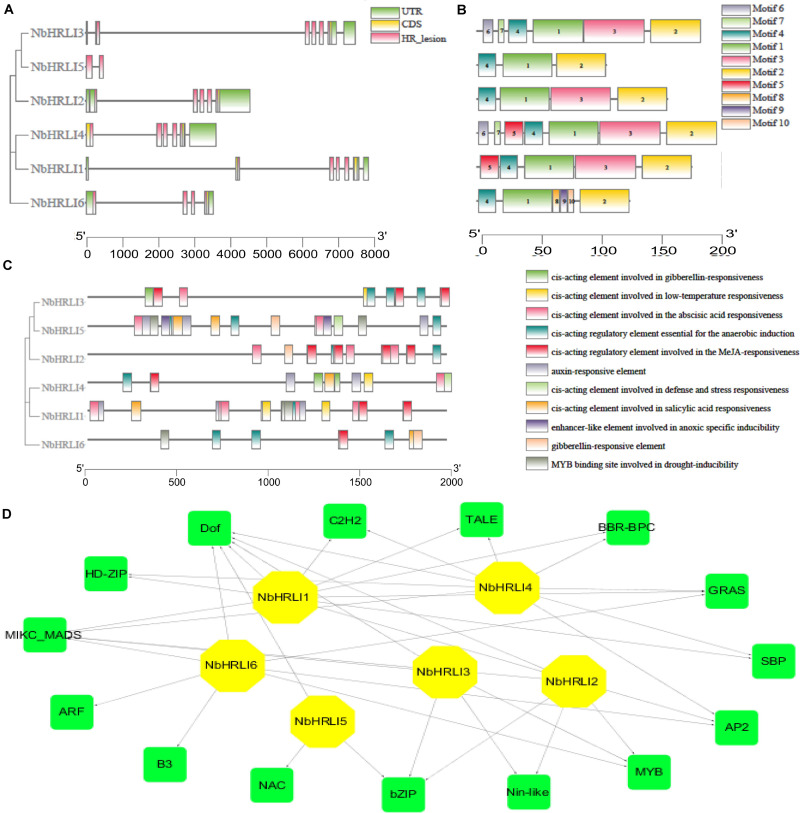
Genome-wide identification and bioinformatic analysis of NbHRLIs. **(A)** Phylogenetic tree of protein sequences and exon-intron structure of NbHRLIs. The HR_lesion domains are shown by pink boxes. Green boxes represent untranslated 5′- and 3′-regions; yellow boxes are exons; black lines represent introns. **(B)** The motif composition of NbHRLIs. The motifs are displayed in different colored boxes. **(C)** Position, quantity, and kind of environmental stress-related and hormone-responsive *cis*-acting elements in NbHRLIs. **(D)** Regulation network between NbHRLIs and potential TFs. Green rectangles represent TFs, yellow hexagons represent NbHRLIs, and black lines indicate potential regulatory relationships.

A genome-wide identification was therefore performed by a two-round Blast against the *N. benthamiana* genome and six NbHRLI proteins were identified (Table 1). Their gene structure and motif composition were analyzed, and the results showed that the *NbHRLIs* are conserved (Figure 1). All NbHRLIs have an HR_lesion domain (Figure 1A) which is conserved in the C-terminus (Supplementary Figure 1). Motif analysis by MEME also showed the conservation of NbHRLIs, because all have motifs 1, 2, and 4, and 4/6 have motif 3 which cover most sections of the protein sequences (Figure 1B and Supplementary Table 3).

**TABLE 1:** General information on HRLI members in *N. benthamiana*.

Gene symbol	Gene locus	Gene position	Strand	CDS (bp)	Protein length (aa)	Theoretical p*I*	Protein MW (kDa)
NbHRLI1	Niben101Scf00737g00006.1	Niben101Scf00737:58266,65947	−	528	175	9.80	19.79
NbHRLI2	Niben101Scf01428g03001.1	Niben101Scf01428:305085,309545	+	468	155	9.90	17.13
NbHRLI3	Niben101Scf01956g10006.1	Niben101Scf01956:1007273,1014598	+	549	182	10.08	20.16
NbHRLI4	Niben101Scf02063g02012.1	Niben101Scf02063:201878,205416	+	474	157	9.32	22.11
NbHRLI5	Niben101Scf03012g00001.1	Niben101Scf03012:50357,50830	+	318	105	10.29	11.52
NbHRLI6	Niben101Scf03570g01013.1	Niben101Scf03570:144139,147604	−	375	124	10.13	13.36

To investigate the regulation network of NbHRLIs, their *cis*-acting elements and potential TFs were predicted by PlantCARE (Lescot et al., 2002) and PlantRegMap (Jin et al., 2017), respectively, using *N. tabacum* as the target species. We focused on the elements related to environmental stress and hormone response, of which the most abundant elements were those involved in SA (7.06%), ABA (23.53%), MeJA (25.88%), auxin (11.76%), and anaerobic induction (15.29%) (Figure 1C and Supplementary Table 4). TFs predicted to regulate over 50% of the NbHRLIs were Dof, MIKC-MADS, GRAS, Myb, AP2, and BZIP (Figure 1D and Supplementary Table 5).

### Expression Pattern of *NbHRLI* Family Members

**FIGURE 2:**
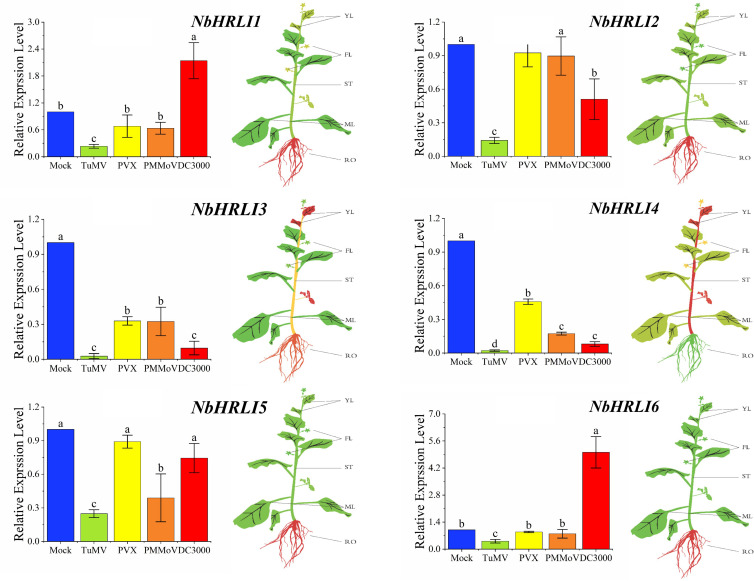
The differential expression of *NbHRLIs* infected with different pathogens and in different tissues by RT-qPCR. YL, young leaf; MF, mature leaf; ST, stem; RO, root; FL, flower. The mean expression values were visualized by TBtools; red represents high expression level and green represents low expression level. *N. benthamiana* was mechanically inoculated with TuMV, PVX, and PMMoV and harvested at 5 dpi. *N. benthamiana* were infiltrated with a suspension of *Pst DC3000* (OD_600_ = 10^–5^) in 10 mM of MgCl_2_ and harvested at 1.5 dpi. The mean expression values were analyzed using *F*-test. Different letters on histograms indicate significant differences (*P* < 0.05).

To better understand the functions of *NbHRLIs*, their expression in different tissues (young leaf, mature leaf, root, flower, and stem) was examined by RT-qPCR. All *NbHRLIs* were expressed less in mature leaves than in young leaves (Figure 2 and Supplementary Figure 2). *NbHRLI5* and *NbHRLI6* had similar expression patterns. Conspicuously, *NbHRLI1/2/3/5/6* were all expressed more highly in roots than *NbHRLI4* while *NbHRLIs 3*/*4* were expressed much more highly in young leaves and stems than the other *NbHRLIs* (Figure 2 and Supplementary Figure 2).

To further investigate whether *NbHRLIs* participate in plant immunity, TuMV, PVX, PMMoV, and the bacterial pathogen *Pseudomonas syringae pv tomato* strain DC3000 (*Pst* DC3000) were inoculated onto young leaves of *N. benthamiana*. The expression levels of all the *NbHRLIs*, and especially *NbHRLI3* and *NbHRLI4*, were reduced by TuMV (Figure 2). The *NbHRLIs* could also be reduced by the other three pathogens, although *NbHRLI1/2/5/6* were not significantly affected in their expression by PVX or PMMoV (although *NbHRLI5* was slightly downregulated by PMMoV). In addition, infection by the bacterial pathogen *Pst* DC3000 increased the expression of *NbHRLI1* and *NbHRLI6*. Thus, most *NbHRLIs* were significantly reduced by TuMV, indicating that they may play roles in the TuMV response.

### Silencing *NbHRLI4* Increases TuMV Accumulation

Because RT-qPCR and transcriptome sequencing had both shown that *Niben101Scf02063g02012.1* (*NbHRLI4*) was strongly reduced by TuMV, we decided to use the TRV-VIGS system to silence *NbHRLI4* and thus investigate its molecular function in response to TuMV. At 10–14 dpi, *NbHRLI4*-silenced plants showed no significant phenotypic change (Supplementary Figure 3A), but the expression of *NbHRLI4* was significantly reduced to 36% of that in the control TRV-00-infected plants. Expression of the other *NbHRLIs* was not significantly altered, suggesting that *NbHRLI4* was specifically silenced (Supplementary Figure 3B).

**FIGURE 3:**
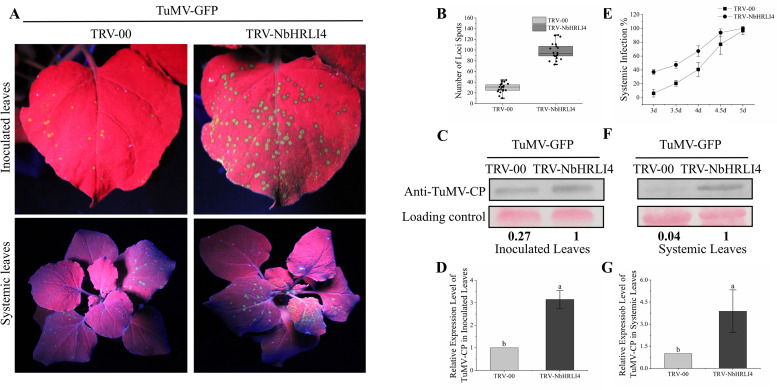
Silencing of *NbHRLI4* induced TuMV accumulation. **(A)** Inoculated and systemic leaves of plants inoculated with TuMV-GFP are observed under UV light. **(B)** Number of infection spots after TuMV infection of leaves inoculated with TRV-00 or TRV-HRLI4 (at least 20 plants per replicate) at 4 dpi. **(C,D)** Western blotting and RT-qPCR showing the increased expression of TuMV-CP **(C)** and mRNA **(D)** in inoculated leaves from TRV-NbHRLI4-infected plants compared with the TRV-00 infected plants at 3 dpi. **(E)** Percentage of plants systemically infected at different times after TuMV inoculation (at least 10 plants per replicate). **(F,G)** Western blotting and RT-qPCR showing the increased expression of TuMV-CP **(F)** and mRNA **(G)** in systemic leaves from TRV-NbHRLI4-infected plants compared with the TRV-00-infected plants at 5 dpi. The mean expression values in this figure were analyzed using *t*-test. Different letters on histograms indicate significant differences (*P* < 0.05).

Subsequently, the *NbHRLI4*-silenced and nonsilenced plants were mechanically inoculated with TuMV-GFP (Figure 3A). At 3 dpi, the numbers of spots (infection foci) on TRV-NbHRLI4-infected plants were nearly three times of those on the nonsilenced (TRV-00-treated) plants (Figure 3B) while RT-qPCR and western blotting analysis showed that TuMV accumulation was greater in the silenced leaves at both the transcriptional and protein levels (Figures 3C,D). Systemic infection also developed more quickly in *NbHRLI4*-silenced plants. At 4 dpi, 41% of nonsilenced control plants had systemic TuMV infection, while 67% of TRV-NbHRLI4-treated plants were systemically infected. At 5 dpi, the figures were respectively 94 and 77% (Figure 3E). Compared with the TRV-00-treated plants, TuMV mRNA and CP accumulation level were both higher in TRV-NbHRLI4-infected systemic leaves (Figures 3F,G). The results therefore show that silencing *NbHRLI4* increased TuMV accumulation.

### Overexpression of NbHRLI4 Reduces TuMV Accumulation

**FIGURE 4:**
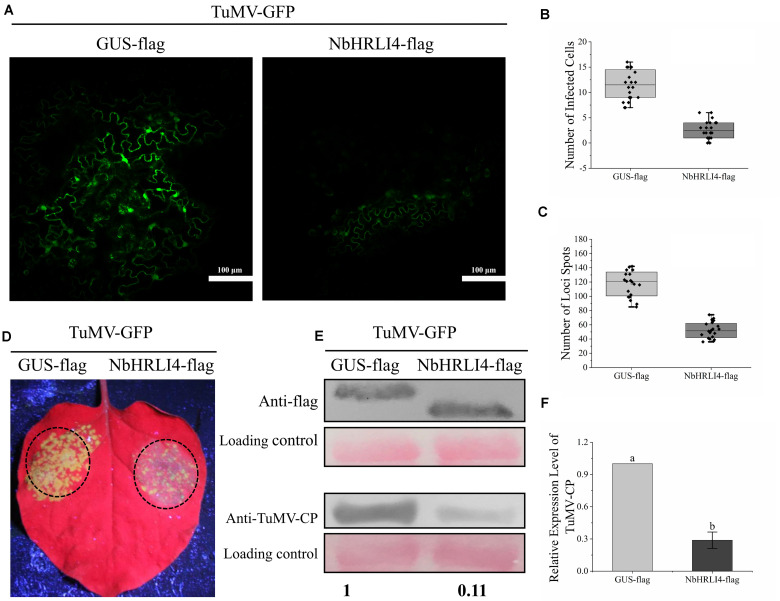
Overexpression of NbHRLI4-reduced TuMV accumulation. **(A)** Green fluorescence observed by laser confocal microscopy in infected cells of leaves overexpressing NbHRLI4-flag or GUS-flag at 72 hpi. Bars = 100 μm. **(B)** Numbers of infected cells in leaves overexpressing NbHRLI4-flag or GUS-flag (at least 20 photos per replicate) determined using the laser confocal microscope at 72 hpi. **(C)** Number of necrotic spots on inoculated leaves overexpressing NbHRLI4-flag or GUS-flag (at least 20 plants per replicate) at 4 dpi. **(D)** Leaves overexpressing NbHRLI4-flag or GUS-flag and inoculated with TuMV-GFP observed under UV light at 4 dpi. **(E)** Western blotting detection of NbHRLI4-flag, GUS-flag, and TuMV-CP at 4 dpi. **(F)** RT-qPCR detection of TuMV mRNA in inoculated leaves from WT plants overexpressing NbHRLI4-flag or GUS-flag. The mean expression values were analyzed using *t*-test. Different letters on histograms indicate significant differences (*P* < 0.05).

NbHRLI4 was then transiently expressed to determine its effect on the TuMV response. The infectious clone TuMV-GFP was coinfiltrated with either PCV-NbHRLI4-flag or PCV-GUS-flag. At 72 hpi, green fluorescence could be observed in infected cells under a laser confocal microscope, but there were fewer infected cells in leaves where NbHRLI4 was expressed compared with the control (GUS-flag) (Figures 4A,B). At 96 hpi, fewer green fluorescent spots were observed in PCV-NbHRLI4-flag-infiltrated leaves under a handheld UV lamp (Figures 4C,D). TuMV mRNA and CP accumulation, detected by RT-qPCR and western blotting, respectively, were much lower in the infiltrated areas of leaves expressing NbHRLI4 than in the controls (Figures 4E,F), confirming that overexpression of NbHRLI4 inhibited TuMV infection.

### NbHRIL4-Induced HR-Like Cell Death in an SA-Dependent Manner

**FIGURE 5:**
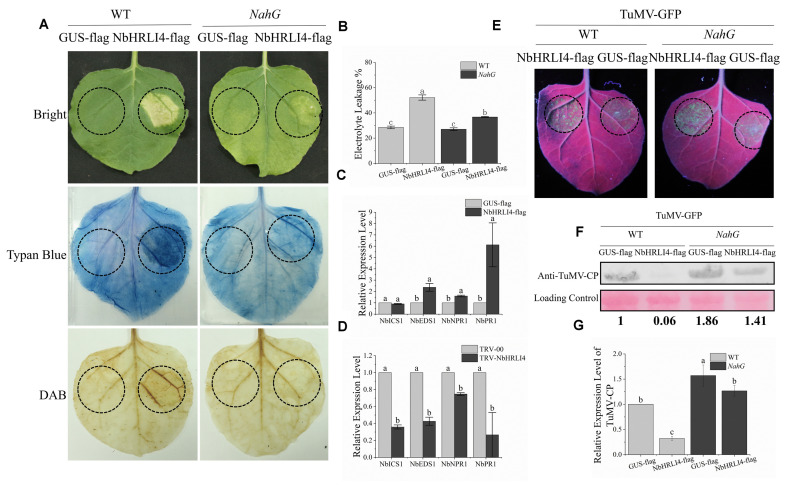
NbHRIL4 induced HR-like cell death in an SA-dependent manner. **(A)** Overexpression of NbHRLI4 (but not GUS) induced cell death (confirmed by trypan blue staining) at 6 dpi. H_2_O_2_ accumulated significantly in areas expressing NbHRLI4 as shown by DAB staining. Overexpression of NbHRLI4 induced less cell death at 6 dpi on *NahG* plants. **(B)** NbHRLI4-flag and GUS-flag were overexpressed in both WT and *NahG* plants. Leaf disks were excised and assayed for electrolyte leakage at 6 dpi. **(C)** Results of RT-qPCR showing the expression levels of *NbEDS1*, *NbICS1*, *NbNPR1*, and *NbPR1* in WT plants overexpressing NbHRLI4-flag or GUS-flag at 3 dpi. **(D)** Results of RT-qPCR showing the expression levels of *NbEDS1*, *NbICS1*, *NbNPR1*, and *NbPR1* in WT plants infected with TRV-HRLI4 or TRV-00 at 12 dpi. *T*-test was used for mean expression values of single gene between two samples in panel **(C)** and panel **(D)**. Different letters on histograms indicate significant differences (*P* < 0.05). **(E)** Leaves of WT and *NahG* plants inoculated with TuMV-GFP and overexpressing NbHRLI4-flag or GUS-flag observed under UV light at 4 dpi. **(F)** Western blotting detection of TuMV-CP on leaves shown in panel **(E)**. **(G)** Results of RT-qPCR to detect TuMV mRNA in leaves shown in panel **(E)**. The mean expression values in panels **(B)** and **(G)** were analyzed using *F*-test. Different letters on histograms indicate significant differences (*P* < 0.05).

HR-like cell death is associated with many defense mechanisms including SA, JA, NO, and ETH. *NbHRLI4* contains the predicted TFs Dof, MIKC-MADS, GRAS, Myb, AP2, and BZIP which have been reported to play roles in the SA pathway (Zhang et al., 2012; Shim et al., 2013; Giri et al., 2014; Dong et al., 2015; Jiang et al., 2016; Shen et al., 2017; Zhou et al., 2018; Yu et al., 2019; Kang and Singh, 2000). To test if *NbHRLI4* was involved in the SA pathway, NbHRLI4-flag was transiently overexpressed by agroinfiltration and HR-like cell death in the infiltrated area was observed. The degree of necrosis increased with the passage of time, and trypan blue staining, H_2_O_2_ assay, and electrolyte leakage assay all confirmed cell death and the accumulation of H_2_O_2_ (Figures 5A,B).

We also examined the relative expression levels of SA-related genes (*NbEDS1*, *NbICS1*, *NbNPR1*, and *NbPR1*) in leaves overexpressing NbHRLI4 and in *NbHRLI4*-silenced plants by RT-qPCR. The expression of all tested genes except *NbICS1* was increased when *NbHRLI4* was overexpressed (Figure 5C) and all were reduced in the silenced plants (Figure 5D).

To further determine whether the cell death was associated with SA, NbHRLI4 was overexpressed in *N. benthamiana* expressing *NahG*. Compared with the WT plants, cell death in *NahG* plants was significantly less at 6 dpi (Figure 5A). The extent of cell death was measured by monitoring electrolyte leakage. In WT plants, electrolyte leakage in the area overexpressing NbHRLI4-flag was 1.83 times that where GUS-flag was overexpressed, but in transgenic *NahG* plants, the ratio was reduced to 1.36 (Figure 5B). Thus, cell death induced by NbHRLI4 was lessened in plants expressing *NahG*, suggesting that SA was involved in this process.

### NbHRLI4-Mediated Immunity Depends on SA

To further study if SA is involved in the NbHRLI4-mediated defense response, we overexpressed PCV-NbHRLI4-flag and PCV-GUS-flag with TuMV-GFP in both WT and *NahG* plants (Figure 5E). Viral RNA and CP protein accumulation in plants overexpressing NbHRLI4 was, respectively, 0.32 and 0.06 times that in WT controls (expressing GUS-flag) and 0.81 and 0.76 times that in *NahG* plants (Figures 5F,G).

**FIGURE 6:**
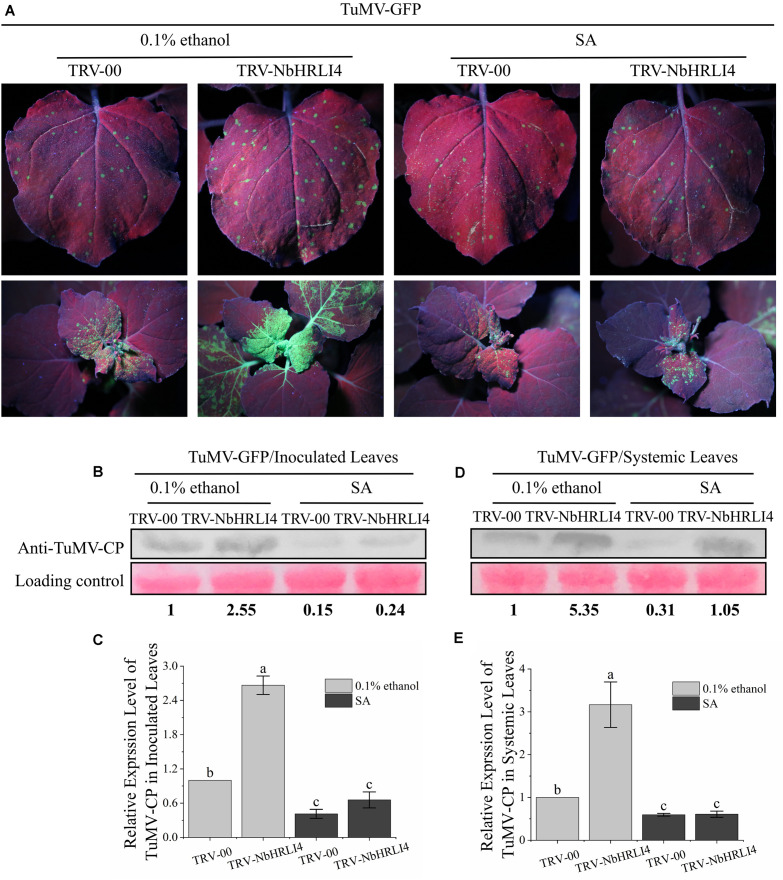
SA is required in NbHRLI4-mediated resistance against TuMV. **(A)** Inoculated and systemic leaves after SA application to plants inoculated with TRV-HRLI4 or TRV-00 (WT) and TuMV-GFP, observed under UV light at 4 dpi. **(B,C)** Western blotting and RT-qPCR detection of TuMV-CP **(B)** and mRNA **(C)** in the inoculated leaves of plants inoculated with TRV-HRLI4 or TRV-00 (WT) and TuMV-GFP and treated with SA or 0.1% ethanol (control). **(D,E)** As panels **(B)** and **(C)** but for the systemic leaves. The mean expression values in this figure were analyzed using *F*-test. Different letters on histograms indicate significant differences (*P* < 0.05).

Exogenous SA (or 0.1% ethanol for the negative control) was then applied to plants inoculated with TRV-00 or TRV-NbHRLI4. Two days after SA application, leaves were mechanically inoculated with TuMV-GFP (Figure 6A). On inoculated leaves where *NbHRLI4* was silenced, SA treatment decreased TuMV-CP accumulation, viral mRNA accumulation, and necrotic spot numbers compared with the controls (Figures 6B,C and Supplementary Figure 4A). Similar results were observed in the systemic leaves (Figures 6D,E and Supplementary Figure 4B). However, SA-treated plants where *NbHRLI4* was silenced were still slightly more susceptible than the nonsilenced (TRV-00-infected) plants (Figure 6), indicating that SA can partially remove susceptibility in TRV-NbHRLI4-infected plants.

The results suggested that the immune response induced by NbHRLI4 depends on SA.

### NbHRLI4 Interacts With TuMV-P3 and Responds to the Expression of TuMV-P3

**FIGURE 7:**
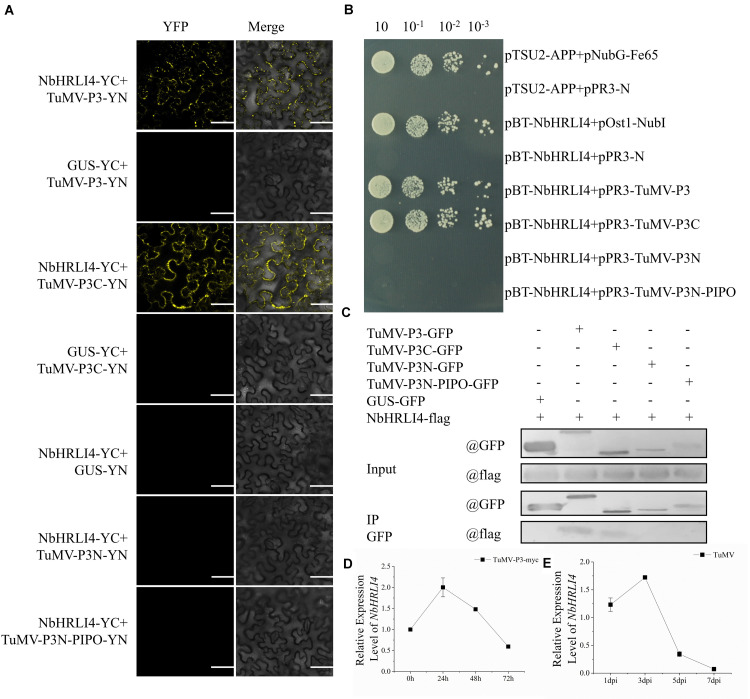
NbHRLI4 interacted with TuMV-P3 and responded to the expression of TuMV-P3. **(A)** BiFC assays were conducted between NbHRLI4/TuMV-P3, NbHRLI4/TuMV-P3C, NbHRLI4/TuMV-P3N, and NbHRLI4/TuMV-P3N-PIPO. GUS was used as negative control. Bars = 50 μm. **(B)** Y2H assays between pBT-NbHRLI4/pPR3-TuMV-P3, pBT-NbHRLI4/pPR3-TuMV-P3C, pBT-NbHRLI4/pPR3-TuMV-P3N, and pBT-NbHRLI4/pPR3-TuMV-P3N-PIPO. pBT-NbHRLI4 with pPR3-N was used as negative control and pBT-NbHRLI4 with pOst1-NubI was the positive control. **(C)** CO-IP assays between NbHRLI4-flag/TuMV-P3-GFP, NbHRLI4-flag/TuMV-P3C-GFP, NbHRLI4-flag/TuMV-P3N-GFP, and NbHRLI4-flag/TuMV-P3N-PIPO-GFP. NbHRLI4-flag with GUS-GFP was the negative control. **(D)** RT-qPCR detection of *NbHRLI4* mRNA in the inoculated leaves at different times (0, 24, 48, and 72 h) of P3-myc expression. **(E)** RT-qPCR detection of NbHRLI4 mRNA in the systemic leaves at different times (1, 3, 5, and 7 days) after TuMV infection.

Previous studies showed that GmHRLI1, a protein in soybean homologous to NbHRLI4, interacts with SMV P3. To test whether NbHRLI4 interacts with TuMV-P3 in our system and to identify the key domain for the interaction, BiFC assays were conducted using the pairs NbHRLI4/TuMV-P3, NbHRLI4/TuMV-P3C, NbHRLI4/TuMV-P3N, and NbHRLI4/TuMV-P3N-PIPO. Yellow fluorescent signals were observed at the cell periphery when NbHRLI4-YC was coexpressed with TuMV-P3-YN or TuMV-P3C-YN (Figure 7A). No fluorescent signals were observed when NbHRLI4-YC was expressed with TuMV-P3N-PIPO-YN or TuMV-P3N-YN or with the control GUS-YN, demonstrating that NbHRLI4 interacts with full-length TuMV-P3 and with its C-terminal portion but not with the N-terminal region of TuMV-P3 or with TuMV-P3N-PIPO. Y2H assays based on the split-ubiquitin system (Figure 7B) and Co-IP assays (Figure 7C) gave similar results.

To further explore the effect of TuMV-P3 on *NbHRLI4*, TuMV-P3-myc and GUS-myc (control) were overexpressed and the levels of *NbHRLI4* mRNA were determined at 24, 48, and 72 hpi. At 24 h, *NbHRLI4* was upregulated nearly two times compared with the control, but expression levels had reduced to ∼50% at 72 hpi (Figure 7D). A similar, but delayed, expression pattern was observed in the systemic (noninoculated) leaves (Figure 7E). The results demonstrated that *NbHRLI4* was upregulated in the early stages of viral P3 expression but downregulated later.

### The Membrane Interaction Domains Are Essential for Interaction With P3 and for HR Induction and Immune Regulation

**FIGURE 8:**
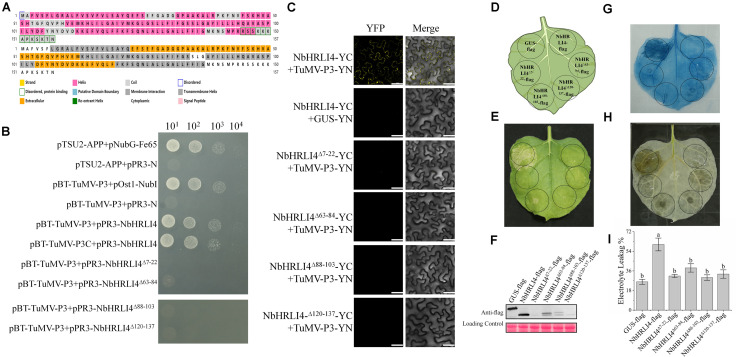
The membrane interaction domains of NbHRLI4 are essential for its interaction with TuMV-P3 and for inducing cell death. **(A)** Protein structure analysis of NbHRLI4. **(B)** Y2H assays between pBT-TuMV-P3/pPR3-NbHRLI4, pBT-TuMV-P3C/pPR3-NbHRLI4, pBT-TuMV-P3/pPR3-NbHRLI4^Δ7–22^, pBT-TuMV-P3/pPR3-NbHRLI4^Δ63–84^, pBT-TuMV-P3/pPR3-NbHRLI4^Δ88–103^, and pBT-TuMV-P3/pPR3-NbHRLI4^Δ120–137^. pBT-TuMV-P3 with pPR3-N was used as negative control and pBT-TuMV-P3 with pOst1-NubI was the positive control. **(C)** BiFC assays were conducted between NbHRLI4/TuMV-P3, NbHRLI4^Δ7–22^/TuMV-P3, NbHRLI4^Δ63–84^/TuMV-P3, NbHRLI4^Δ88–103^/TuMV-P3, and NbHRLI4^Δ120–137^/TuMV-P3. GUS was used as negative control. Bars = 50 μm. **(D)** Schematic diagram of transient infiltration of GUS-flag, NbHRLI4-flag, NbHRLI4^Δ7–22^-flag, NbHRLI4^Δ63–84^-flag, NbHRLI4^Δ88–103^-flag, and NbHRLI4^Δ120–137^-flag in panels **(E–G)**. **(E)** Overexpression of GUS-flag, NbHRLI4-flag, NbHRLI4^Δ7–22^-flag, NbHRLI4^Δ63–84^-flag, NbHRLI4^Δ88–103^-flag, and NbHRLI4^Δ120–137^-flag in bright at 6 dpi. **(F)** Western blotting detection of proteins expressed on leaves shown in panel **(D)**. **(G)** Trypan blue staining of leaves expressing the vectors in panel **(E)** at 6 dpi. **(H)** DAB staining of leaves expressing the vectors in panel **(E)** at 6 dpi. **(I)** Leaf disks expressing the vectors in panel **(E)** were excised and assayed for electrolyte leakage at 6 dpi. The mean expression values were analyzed using *F*-test. Different letters on histograms indicate significant differences (*P* < 0.05).

To further investigate the biological significance of the interaction between TuMV P3 protein and NbHRLI4, we analyzed the structure of NbHRLI4 and identified four membrane interaction domains (Figure 8A). Y2H and BiFC assays using deletion mutants of NbHRLI4 showed that all the domains were necessary for interaction with TuMV-P3 (Figures 8B,C).

**FIGURE 9:**
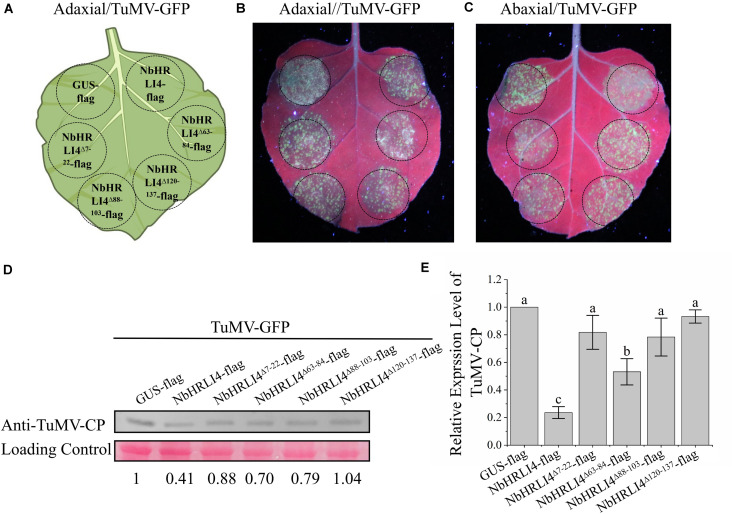
The membrane interaction domains of NbHRLI4 are essential for resistance to TuMV. **(A)** Schematic diagram showing transient infiltration of GUS-flag, NbHRLI4-flag, NbHRLI4^Δ7–22^-flag, NbHRLI4^Δ63–84^-flag, NbHRLI4^Δ88–103^-flag, and NbHRLI4^Δ120–137^-flag combined with TuMV in panels **(B,C)**. **(B)** Adaxial surface of leaves expressing the vectors in panel **(A)** under UV light at 4 dpi. **(C)** Abaxial surface of leaves expressing the vectors in panel **(A)** under UV light at 4 dpi. **(D)** Western blotting detection of TuMV-CP on leaves shown in panels **(B,C)**. **(E)** Results of RT-qPCR to detect TuMV mRNA in leaves shown in panels **(B,C)**. The mean expression values were analyzed using *F*-test. Different letters on histograms indicate significant differences (*P* < 0.05).

We also constructed vectors to overexpress each of the single domain deletion mutants, NbHRLI4^Δ7–22^-flag, NbHRLI4^Δ63–84^-flag, NbHRLI4^Δ88–103^-flag, and NbHRLI4^Δ120–137^-flag (Figures 8D–F). Overexpression of NbHRLI4^Δ63–84^-flag induced less HR than the full-length NbHRLI4-flag, and mutants of the other three domains did not induce HR or H_2_O_2_ accumulation (Figures 8G,H). Consistently, electrolyte leakage in areas injected with any of the four mutants was lower than that in areas expressing NbHRLI4-flag (Figure 8I). When TuMV-GFP was coexpressed with any of these mutants, TuMV accumulation was significantly greater than in the NbHRLI4+TuMV-GFP control (Figure 9).

These results show that all four membrane interaction domains are necessary for interaction with TuMV-P3, for HR induction, and for resistance to TuMV.

### Relationship Between NbHRLI4 and NbHIR3s

In our previous study, we showed that NbHIR3s induced HR in an SA-dependent manner (Li S. et al., 2019), and this resembles what we have now found with NbHRLI4. However, when TRV-based knockdown of each gene was combined with ectopic overexpression analyses, there were no significant differences in the degree of HR, suggesting that NbHRLI4 and NbHIR3s regulate cell death by independent pathways (Supplementary Figure 5).

## Discussion

*HRLIs* have rarely been characterized, but this protein family may have important roles in HR-like cell death. In this study, six *HRLIs* were characterized in *N. benthamiana*, a plant widely used to study plant-pathogen interactions. These NbHRLIs are closely related to one another and have an HR_lesion domain in the C-terminal region (Figure 1A).

HR-like cell death is necessarily a strictly regulated process since excessive cell death damages plant development (Lam, 2004). Therefore, the number of NbHRLI family members is relatively small compared with other gene families in *N. benthamiana*. When infected with TuMV, young leaves were collected to detect relative gene expression at 6 dpi. All *NbHRLIs* were downregulated but especially *NbHRLI3* and *NbHRLI4*, indicating that *NbHRLIs* may be involved in the response to TuMV infection (Figure 2). *Niben101Scf02063g02012.1* (*NbHRLI4*) was selected for further functional analysis. Meanwhile, we investigated the role of *NbHRLI3* in TuMV infection (Supplementary Figures 6, 7). *NbHRLI3* has a similar function as *NbHRLI4*, indicating a conserved role of *NbHRLIs*.

Despite its significant downregulation later, *NbHRLI4* had a nearly twofold upregulation in the early infection stage, and it was also initially upregulated when TuMV-P3 was expressed. An interaction between NbHRLI4 and TuMV-P3 was confirmed, and the C-terminal region of P3 was shown to be essential for the interaction (Figure 7). A similar pattern was reported from a different potyvirus/host combination (SMV-soybean), in which *GmHRLI1* was upregulated in the early stages (12 hpi) of infection and GmHRLI1 interacted with SMV-P3 (Luan et al., 2019).

The SA pathway plays a key role in inducing cell death and basal defense response against various pathogens (Baebler et al., 2014; Hwang et al., 2014; Xu et al., 2018; Miao et al., 2019). Several host proteins are known to be involved in the SA pathway in response to TuMV infection. For example, plants with lower levels of guanosine tetraphosphate and pentaphosphate [(p)ppGpp] had higher resistance to TuMV, and this was associated with an increased SA content and expression of SA-related genes (Abdelkefi et al., 2018). In addition, we have previously reported that NbALD1 mediates a defense response against TuMV by the SA pathway (Wang et al., 2019). AtCA1 is also a mediator of SA defense responses. TuMV HCPro interacts with AtCA1, compromising the SA pathway and weakening resistance (Poque et al., 2018). Similar to our findings, TuMV induced *NbALD1* or *AtCA1* upregulation in the early stages of infection, and silencing or mutation of the two genes made plants more susceptible to TuMV.

However, unlike NbHRLI4, AtCA1 and NbALD1 do not induce HR-like cell death. SA can affect three main stages of the virus infection cycle: intercellular trafficking, long-distance movement, and replication, and therefore not all genes depending on the SA pathway can induce HR (Alazem and Lin, 2015; Collum and Culver, 2016; Palukaitis et al., 2017). However, there are many studies of genes that do induce cell death and inhibit virus in SA-mediated ways, including rgs-CaM and Ny-1 (Baebler et al., 2014; Jeon et al., 2017). SA-mediated plant defense is a complex process and does not only operate by inducing cell death; other molecular events including the expression of PR-related genes and callose deposition can also contribute to the antiviral effect (Baebler et al., 2014; Alazem and Lin, 2015; Zhao and Li, 2021). There are also other studies suggesting that virus resistance is induced through unknown pathways independent from cell death (Komatsu et al., 2010). Although cell death occurred in this study, we cannot exclude the contribution of other molecular events to TuMV defense. However, we tend to believe that the resistance induced by NbHRLI4 is mainly caused by cell death because we mutated four membrane domains and HR induced by NbHRLI4 was weakened along with weakened resistance.

NbHIR3s induce HR in a SA-dependent manner (Li S. et al., 2019), which is similar to what we now report for NbHRLI4. However, it appears that these proteins may regulate cell death by independent pathways (Supplementary Figure 7). Moreover, *NbEDS1* is required for *NbHIR3s* to induce HR (Li S. et al., 2019), but when the SA pathway genes *NbEDS1*, *NbICS1*, *NbNPR1*, and *NbPR1* were silenced, overexpression of NbHRLI4 still induced HR (data not shown). Also, whereas overexpression of NbHIR3s in *NahG* plants did not induce HR, transient expression of NbHRLI4 in *NahG* plants in this study still induced some cell death, although significantly less than that in WT plants. It therefore seems that the specific components in the SA pathway regulating HR induced by NbHIR3s differ from those induced by NbHRLI4. Crosstalk between SA and other pathways involved in defense responses is common (Jiang et al., 2016; Li N. et al., 2019; Wang et al., 2019; Yang J. et al., 2019) and needs further study to elucidate the precise pathway involved in NbHRLI4-mediated cell death.

Taken together, the results here indicate that NbHRLI4 regulates the SA-dependent pathway to induce cell death and participates in defense against TuMV.

## Data Availability Statement

The original contributions presented in the study are included in the article/Supplementary Material, further inquiries can be directed to the corresponding authors.

## Author Contributions

XW, YCL, and HZ initiated and designed the experiment. XW, YCL, LQL, SR, MJ, KH, JW, YWL, JP, LL, and GW performed the experiments and collected the data. XW and FY analyzed the data and wrote the manuscript. HZ, FY, and JC revised the manuscript. All authors read and approved the final manuscript.

## Conflict of Interest

The authors declare that the research was conducted in the absence of any commercial or financial relationships that could be construed as a potential conflict of interest.

## Supplementary Material

Supplementary Figure 1 Multiple sequence alignment of the NbHRLIs created by DNAMAN.

Supplementary Figure 2 The differential expression of *NbHRLI* genes in different tissues by RT-qPCR (raw data). YL, young leaf; MF, mature leaf; ST, stem; RO, root; FL, flower. The mean expression values were calculated from three independent biological replicates using *F*-test and are relative to that in young leaves. Different letters on histograms indicate significant differences (*P* < 0.05).

Supplementary Figure 3 Effect of TRV-induced *NbHRLI4* silencing on *N. benthamiana*. **(A)** *NbHRLI4* silencing caused no significant phenotypic change at 12 dpi. **(B)** Detection of *NbHRLI* transcripts in TRV-NbHRLI4-infected plants by RT-qPCR. The mean expression values were analyzed using *F*-test. Different letters on histograms indicate significant differences (*P* < 0.05).

Supplementary Figure 4 Number of necrotic spots and percentage of plants systemically infected when WT plants were infected with TRV-NbHRLI4 or TRV-00 and treated with SA or 0.1% ethanol. **(A)** Number of necrotic spots at 4 dpi (at least 20 plants per replicate). **(B)** Percentage of plants systemically infected at different times after TuMV inoculation (at least 10 plants per replicate).

Supplementary Figure 5 The cell death induced by NbHRLI4 or NbHIR3s were independent of each other. **(A)** Silencing of *NbHRLI4* or *NbHIR3s* caused no significant phenotypic change at 12 dpi. **(B,C)** Detection of the transcripts of *NbHIR3s* **(B)** and *NbHRLI4* **(C)** in TRV- 00-, TRV- NbHRLI4-, and TRV-NbHIR3-infected plants by RT-qPCR. The mean expression values were analyzed using *F*-test. Different letters on histograms indicate significant differences (*P* < 0.05). **(D)** Overexpression of GUS-flag, NbHIR3.1-flag in *NbHRLI4*-silenced plants as well as overexpression of GUS-flag, NbHRLI4-flag in *NbHIR3-*silenced plants in bright light at 6 dpi. **(E)** DAB staining of leaves in panel **(D)** at 6 dpi.

Supplementary Figure 6 Silencing of *NbHRLI3* induced TuMV accumulation. **(A)** RT-qPCR detection of NbHRLI4 mRNA in the systemic leaves at different times (1, 3, 5, and 7 days) after TuMV infection. **(B)** Detection of *NbHRLI* transcripts in TRV-NbHRLI3-infected plants by RT-qPCR. The mean expression values were analyzed using *F*-test. Different letters on histograms indicate significant differences (*P* < 0.05). **(C)** *NbHRLI4* silencing caused no significant phenotypic change at 12 dpi. **(D)** Inoculated and systemic leaves of plants inoculated with TuMV-GFP were observed under UV light. **(E,F)** Western blotting and RT-qPCR showing the increased expression of TuMV-CP **(E)** and mRNA **(F)** in inoculated leaves from TRV-NbHRLI3-infected plants compared with the TRV-00-infected plants at 3 dpi. **(G,H)** Western blotting and RT-qPCR showing the increased expression of TuMV-CP **(G)** and mRNA **(H)** in systemic leaves from TRV-NbHRLI3-infected plants compared with the TRV-00-infected plants at 5 dpi. The mean expression values were analyzed using *t*-test. Different letters on histograms indicate significant differences (*P* < 0.05).

Supplementary Figure 7 Overexpression of NbHRLI3 induced HR-like cell death and reduced TuMV accumulation. **(A)** Overexpression of NbHRLI3 (but not GUS) induced cell death at 6 dpi. **(B)** H_2_O_2_ accumulated significantly in areas expressing NbHRLI3 as shown by DAB staining. **(C)** NbHRLI3-flag and GUS-flag were overexpressed, and leaf disks were excised and assayed for electrolyte leakage at 6 dpi. **(D)** Leaves overexpressing NbHRLI3-flag or GUS-flag and inoculated with TuMV-GFP were observed under UV light at 4 dpi. **(E)** Western blotting detection of NbHRLI3-flag, GUS-flag, and TuMV-CP at 4 dpi. **(F)** RT-qPCR detection of TuMV mRNA in inoculated leaves from plants overexpressing NbHRLI3-flag or GUS-flag. The mean expression values were analyzed using *F*-test. Different letters on histograms indicate significant differences (*P* < 0.05).

Supplementary Table 1 Information of transcriptome sequence.

Supplementary Table 2 Primers used in this study.

Supplementary Table 3 Differentially expressed HR-related genes in TuMV-infected *N. benthamiana* detected by transcriptome sequencing.

Supplementary Table 4 The MEME motif sequence and length of NbHRLIs.

Supplementary Table 5 *Cis*-acting elements in *NbHRLIs*.

Supplementary Table 6 Potential transcription factors of *NbHRLIs.*

Supplementary Table 7 Constructs used in Figures 7, 8.

## Abbreviations

HR: hypersensitive response
HRLI: HR-like lesion-inducing protein
TuMV: turnip mosaic virus
PVX: potato virus X
SA: salicylic acid
SMV: soybean mosaic virus
WT: wild type
TRV: tobacco rattle virus
VIGS: virus induced gene silencing
dpi: days post-inoculation
hpi: hours post infiltration
Y2H: yeast two-hybrid
BiFC: bimolecular fluorescence complementation
TFs: transcription factors
ABA: abscisic acid
MeJA: methyl jasmonate
PMMoV: pepper mottle mosaic virus.
