# Supplementary figures and images for: Genome-Wide Identification Reveals That Nicotiana benthamiana Hypersensitive Response (HR)-Like Lesion Inducing Protein 4 (NbHRLI4) Mediates Cell Death and Salicylic Acid-Dependent Defense Responses to Turnip Mosaic Virus
Source: Front Plant Sci. 2021 May 25;12:627315. doi: 10.3389/fpls.2021.627315 (PMC8185164; doi:10.3389/fpls.2021.627315)

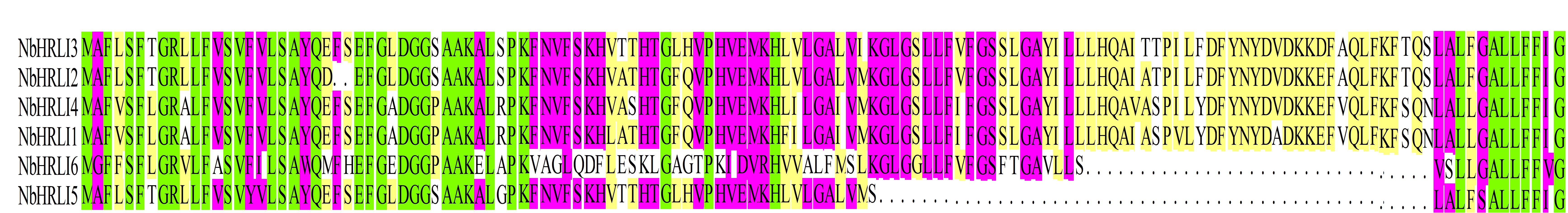

Supplement: Supplementary Figure 1 — Multiple sequence alignment of the NbHRLIs created by DNAMAN. [file Image_1.JPEG]

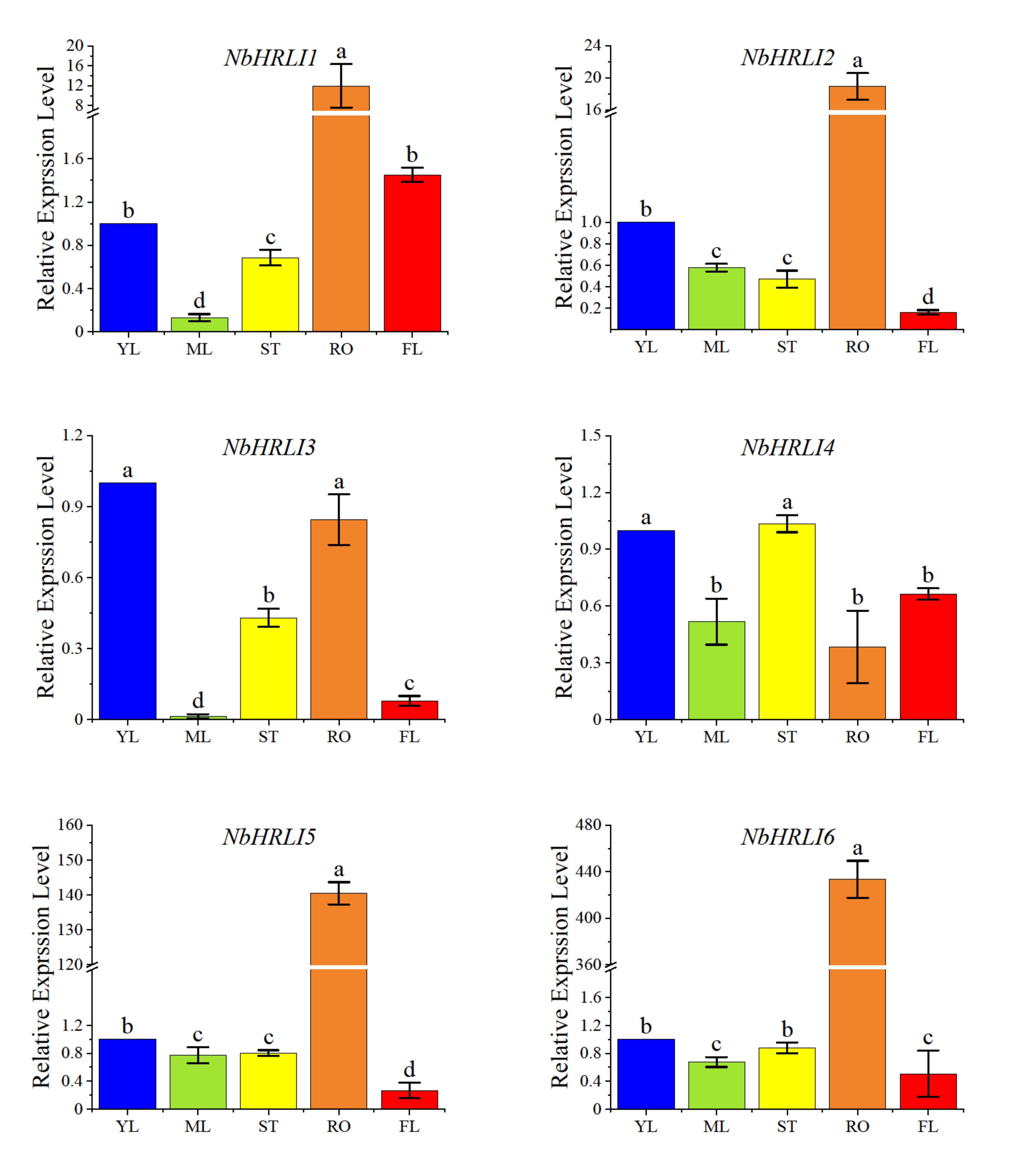

Supplement: Supplementary Figure 2 — The differential expression of NbHRLI genes in different tissues by RT-qPCR (raw data). YL, young leaf; MF, mature leaf; ST, stem; RO, root; FL, flower. The mean expression values were calculated from three independent biological replicates using F-test and are relative to that in young leaves. Different letters on histograms indicate significant differences (P < 0.05). [file Image_2.JPEG]

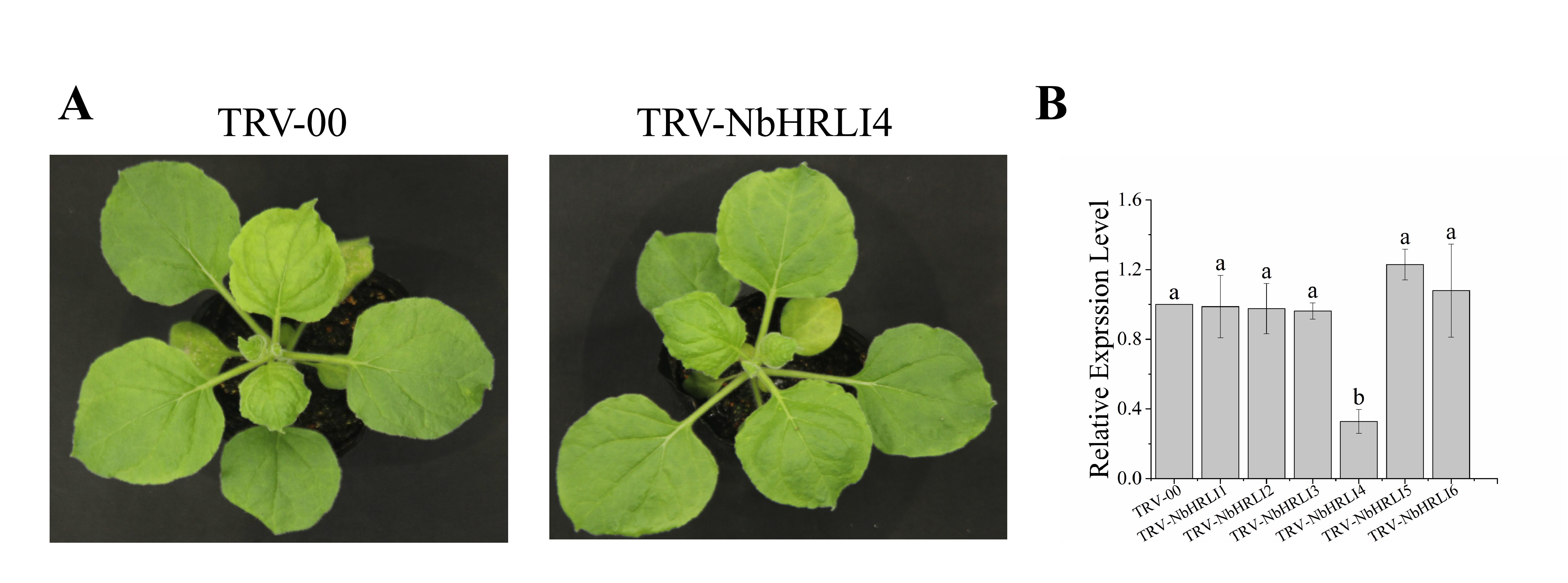

Supplement: Supplementary Figure 3 — Effect of TRV-induced NbHRLI4 silencing on N. benthamiana. (A) NbHRLI4 silencing caused no significant phenotypic change at 12 dpi. (B) Detection of NbHRLI transcripts in TRV-NbHRLI4-infected plants by RT-qPCR. The mean expression values were analyzed using F-test. Different letters on histograms indicate significant differences (P < 0.05). [file Image_3.JPEG]

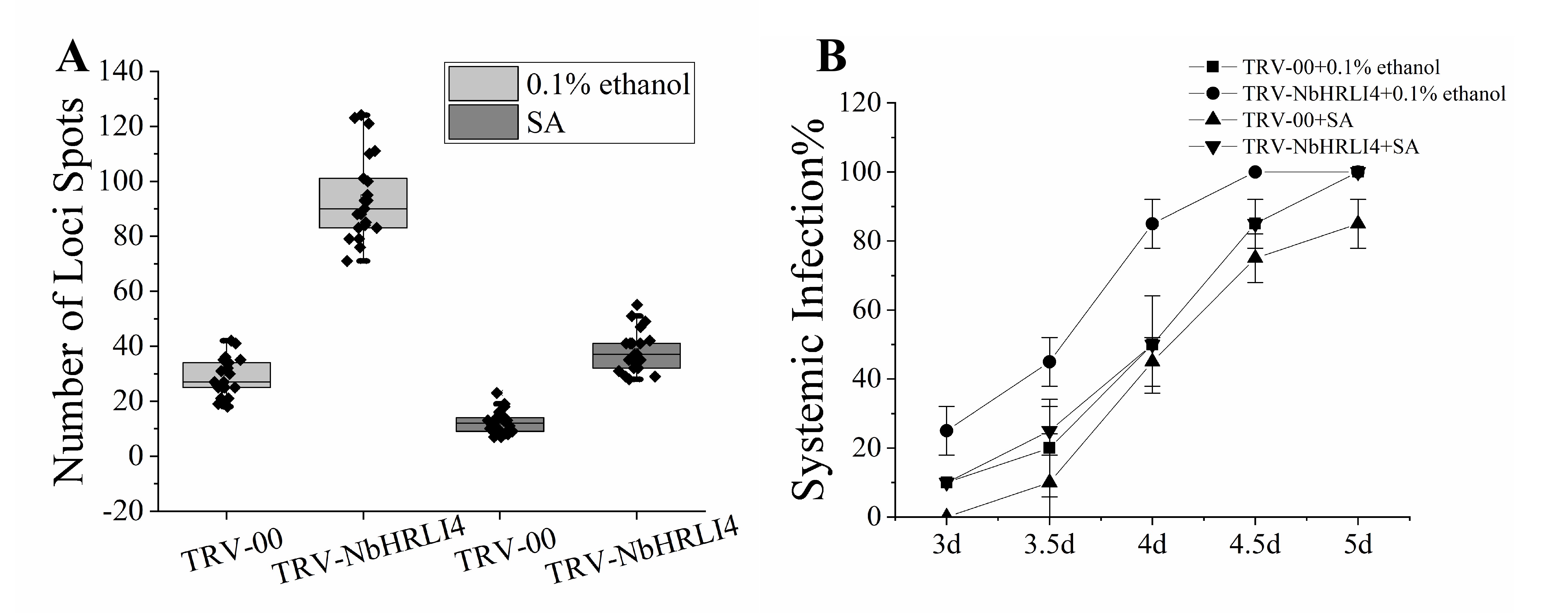

Supplement: Supplementary Figure 4 — Number of necrotic spots and percentage of plants systemically infected when WT plants were infected with TRV-NbHRLI4 or TRV-00 and treated with SA or 0.1% ethanol. (A) Number of necrotic spots at 4 dpi (at least 20 plants per replicate). (B) Percentage of plants systemically infected at different times after TuMV inoculation (at least 10 plants per replicate). [file Image_4.JPEG]

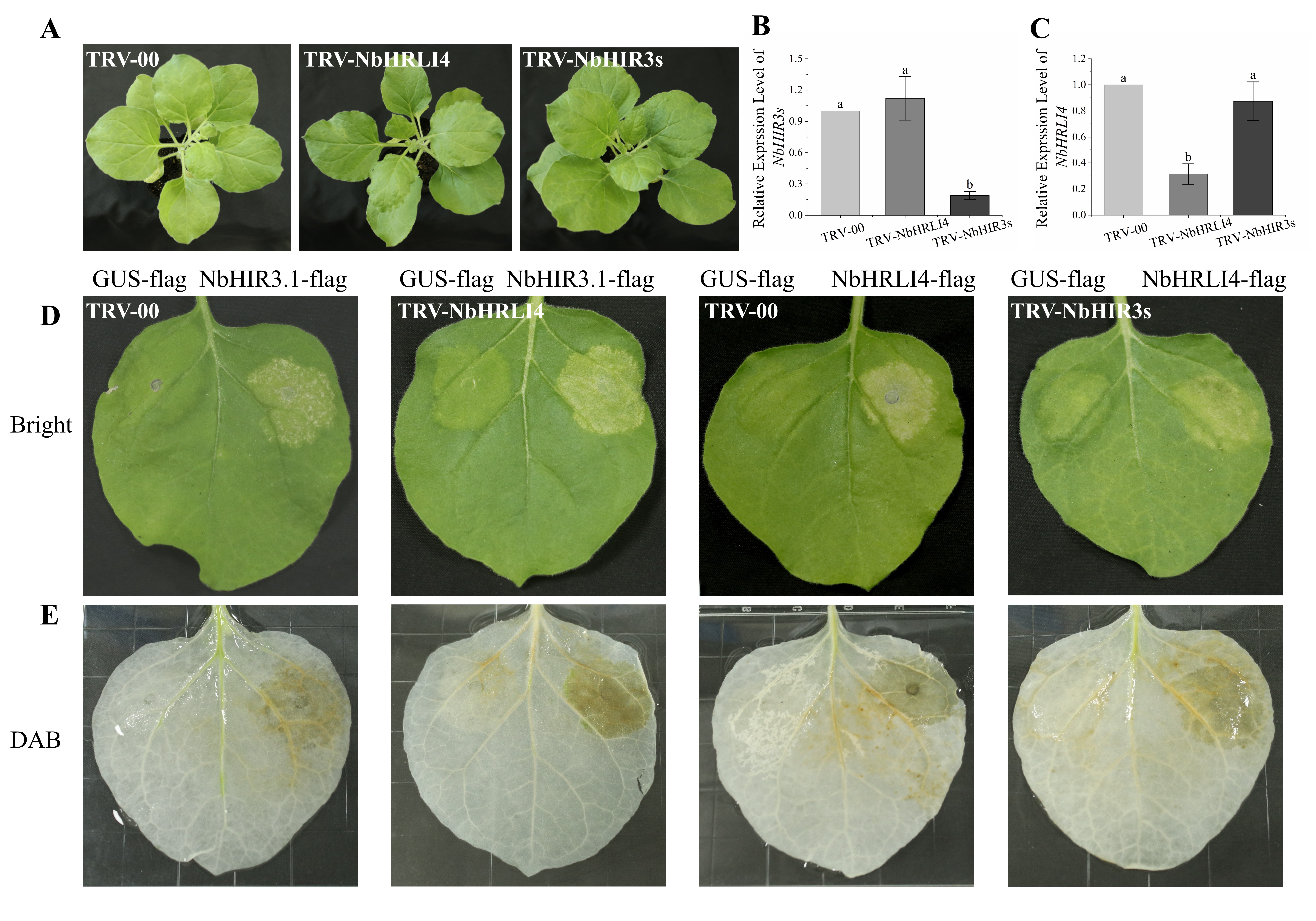

Supplement: Supplementary Figure 5 — The cell death induced by NbHRLI4 or NbHIR3s were independent of each other. (A) Silencing of NbHRLI4 or NbHIR3s caused no significant phenotypic change at 12 dpi. (B,C) Detection of the transcripts of NbHIR3s (B) and NbHRLI4 (C) in TRV- 00-, TRV- NbHRLI4-, and TRV-NbHIR3-infected plants by RT-qPCR. The mean expression values were analyzed using F-test. Different letters on histograms indicate significant differences (P < 0.05). (D) Overexpression of GUS-flag, NbHIR3.1-flag in NbHRLI4-silenced plants as well as overexpression of GUS-flag, NbHRLI4-flag in NbHIR3-silenced plants in bright light at 6 dpi. (E) DAB staining of leaves in panel (D) at 6 dpi. [file Image_5.JPEG]

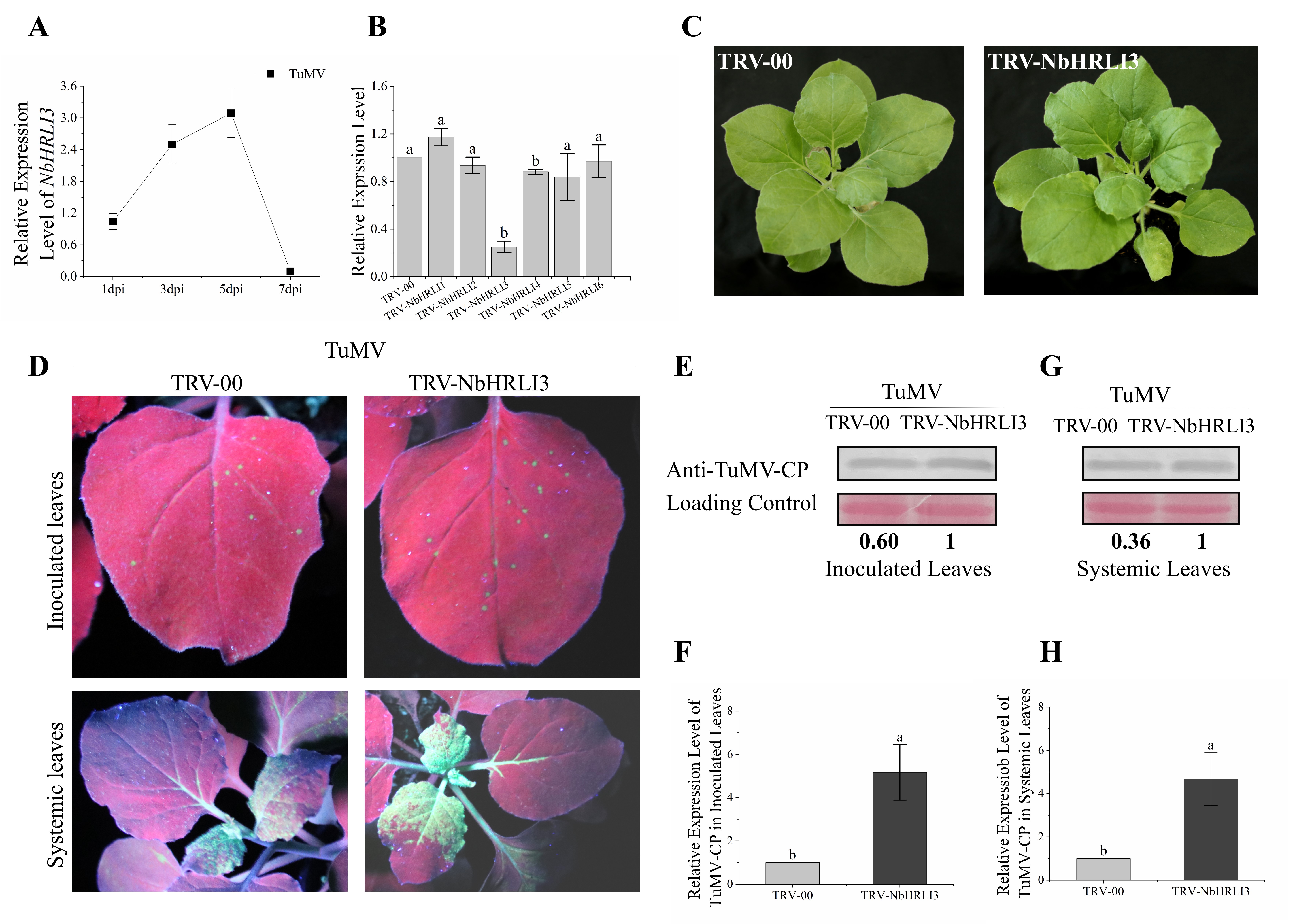

Supplement: Supplementary Figure 6 — Silencing of NbHRLI3 induced TuMV accumulation. (A) RT-qPCR detection of NbHRLI4 mRNA in the systemic leaves at different times (1, 3, 5, and 7 days) after TuMV infection. (B) Detection of NbHRLI transcripts in TRV-NbHRLI3-infected plants by RT-qPCR. The mean expression values were analyzed using F-test. Different letters on histograms indicate significant differences (P < 0.05). (C) NbHRLI4 silencing caused no significant phenotypic change at 12 dpi. (D) Inoculated and systemic leaves of plants inoculated with TuMV-GFP were observed under UV light. (E,F) Western blotting and RT-qPCR showing the increased expression of TuMV-CP (E) and mRNA (F) in inoculated leaves from TRV-NbHRLI3-infected plants compared with the TRV-00-infected plants at 3 dpi. (G,H) Western blotting and RT-qPCR showing the increased expression of TuMV-CP (G) and mRNA (H) in systemic leaves from TRV-NbHRLI3-infected plants compared with the TRV-00-infected plants at 5 dpi. The mean expression values were analyzed using t-test. Different letters on histograms indicate significant differences (P < 0.05). [file Image_6.JPEG]

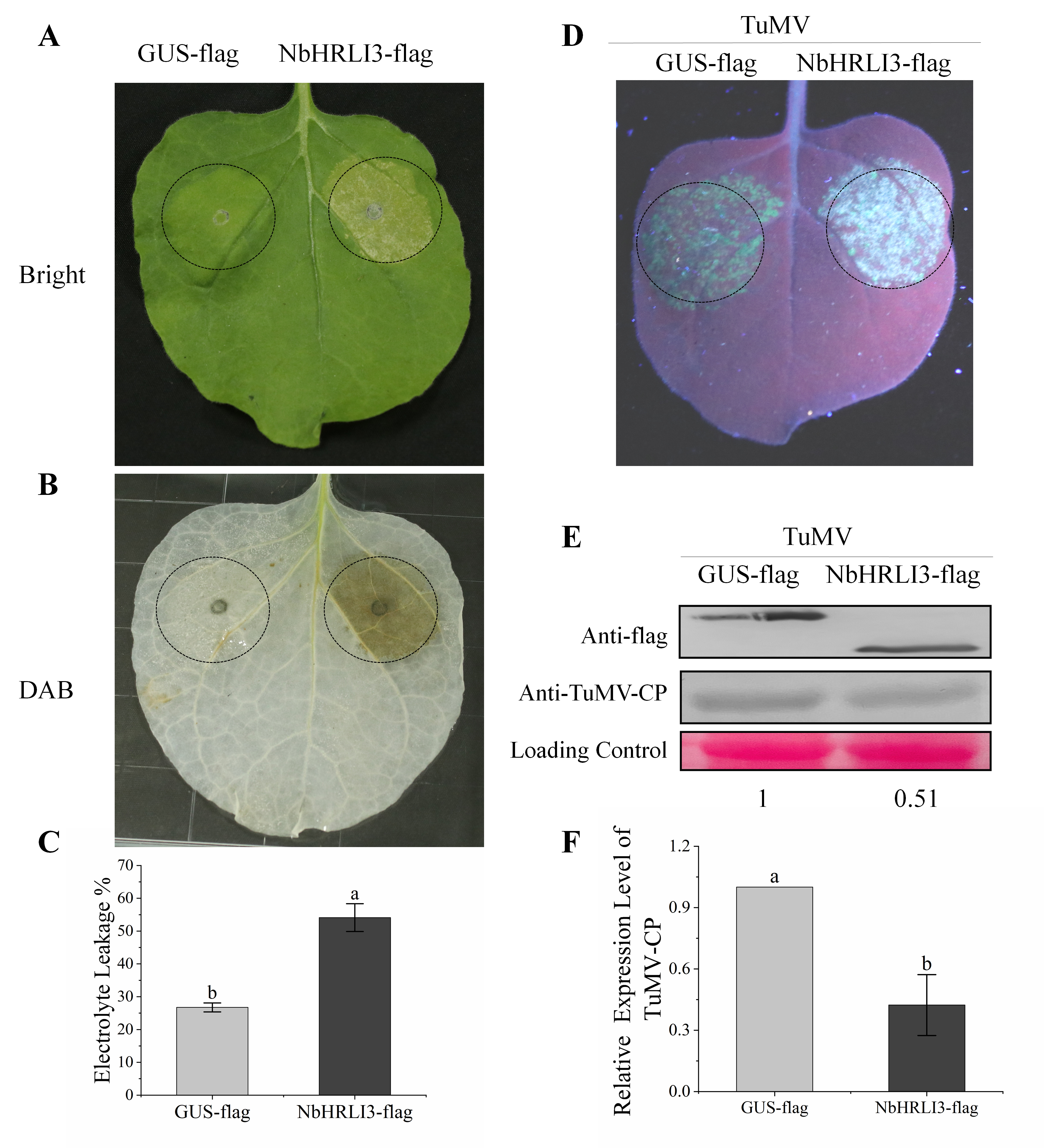

Supplement: Supplementary Figure 7 — Overexpression of NbHRLI3 induced HR-like cell death and reduced TuMV accumulation. (A) Overexpression of NbHRLI3 (but not GUS) induced cell death at 6 dpi. (B) H2O2 accumulated significantly in areas expressing NbHRLI3 as shown by DAB staining. (C) NbHRLI3-flag and GUS-flag were overexpressed, and leaf disks were excised and assayed for electrolyte leakage at 6 dpi. (D) Leaves overexpressing NbHRLI3-flag or GUS-flag and inoculated with TuMV-GFP were observed under UV light at 4 dpi. (E) Western blotting detection of NbHRLI3-flag, GUS-flag, and TuMV-CP at 4 dpi. (F) RT-qPCR detection of TuMV mRNA in inoculated leaves from plants overexpressing NbHRLI3-flag or GUS-flag. The mean expression values were analyzed using F-test. Different letters on histograms indicate significant differences (P < 0.05). [file Image_7.JPEG]
