# Supplementary material for: Genome-Wide Identification Reveals That Nicotiana benthamiana Hypersensitive Response (HR)-Like Lesion Inducing Protein 4 (NbHRLI4) Mediates Cell Death and Salicylic Acid-Dependent Defense Responses to Turnip Mosaic Virus
Source: Front Plant Sci. 2021 May 25;12:627315. doi: 10.3389/fpls.2021.627315 (PMC8185164; doi:10.3389/fpls.2021.627315)
Supplement: Supplementary Table 1 — Information of transcriptome sequence. [file Table_1.DOCX]

**Table S1 Information of transcriptome sequence**

| **Extract Protocol** | Total RNA was extracted using Trizol reagent (Invitrogen, CA, USA) following the manufacturer’s procedure. The total RNA quantity and purity were analysis of Bioanalyzer 2100 and RNA 6000 Nano LabChip Kit (Agilent, CA, USA) with RIN number >7.0. Approximately 10 μg of total RNA representing a specific adipose type was subjected to isolate Poly (A) mRNA with poly-T oligo-attached magnetic beads (Invitrogen). Following purification, the poly(A)- or poly(A)+ RNA fractions is fragmented into small pieces using divalent cations under elevated temperature. Then the cleaved RNA fragments were reverse-transcribed to create the final cDNA library in accordance with the protocol for the mRNA-Seq sample preparation kit (Illumina, San Diego, USA), the average insert size for the paired-end libraries was 300 bp (±50 bp). And then we performed the paired-end sequencing on an Illumina Novaseq™ 6000 at the (lc-bio, China) following the vendor's recommended protocol. |
| --- | --- |
| **Library Construction Protocol** | RNA libraries were prepared for sequencing using standard Illumina protocols |
| **Library Strategy** | RNA-seq |
| **Data Processing Step** | Illumina Casava1.7 software used for basecalling. |
| **Data Processing Step** | Sequenced reads were trimmed for adaptor sequence, and masked for low-complexity or low-quality sequence, then mapped to mm8 whole genome using bowtie v0.12.2 with parameters -q -p 4 -e 100 -y -a -m 10 --best --strata |
| **Data Processing Step** | Reads Per Kilobase of exon per Megabase of library size (FPKM) were calculated using a protocol from Chepelev et al., Nucleic Acids Research, 2009. In short, exons from all isoforms of a gene were merged to create one meta-transcript. The number of reads falling in the exons of this meta-transcript were counted and normalized by the size of the meta-transcript and by the size of the library. |
| **Data Processing Step** | Data filtering steps.Prior to assembly, the low quality reads(1,reads containing sequencing adaptors; 2,reads containing sequencing primer;3, nucleotide with q quality score lower than 20 )were removed .software CutAdapter,parameters -o 5 -p 100 |
| **Data Processing Step** | Read alignment software,software HISAT ,version 2.0 ,parameters -l fr-firststrand -mi 20 -mx 500000 -p 2 -b dta -q phred33-quals -x 9 |
| **Data Processing Step** | Perform expression level for mRNAs by calculating FPKM.stimate the expression levels of all transcripts.software StringTie ,version 1.3.0,parameters -b dta -q phred33-quals. |
| **Genome Build** | Beta_vulgaris.GCF_000511025.2_RefBeet-1.2.2 |
| **Processed Data Files Format and Content** | Tab-delimited text files include FPKM values for each Sample |
