# Supplementary material for: Genome-Wide Identification Reveals That Nicotiana benthamiana Hypersensitive Response (HR)-Like Lesion Inducing Protein 4 (NbHRLI4) Mediates Cell Death and Salicylic Acid-Dependent Defense Responses to Turnip Mosaic Virus
Source: Front Plant Sci. 2021 May 25;12:627315. doi: 10.3389/fpls.2021.627315 (PMC8185164; doi:10.3389/fpls.2021.627315)
Supplement: Supplementary Table 2 — Primers used in this study. [file Table_2.DOC]

Table S2 Primers used in this study

| Primers | Sequence |
| --- | --- |
| NbDLUBC-RT-F | TTTCGGTCCTGATGATACTCCC |
| NbDLUBC-RT-R | CACAGAGCAAAGACTGGATTGA |
| NbICS1-RT-F | AAGGAAGTTCAGTTATTGCTGC |
| NbICS1-RT-R | GCTTGGTCGTAAGATGCTTTA |
| NbEDS1-RT-F | GGTGAACGCTCTGAGTCTTG |
| NbEDS1-RT-R | CATTCTGTGCTGAGTAGGGAG |
| NbNPR1-RT-F | TGAGATTCTGGAGCAAGCA |
| NbNPR1-RT-R | GTTGTCCTCTGTGCGTTGA |
| NbPR1-RT-F | GATGCCCATAACACAGCTCG |
| NbPR1-RT-R | TCGCCGTATTGACCATGAGA |
| NbHRLI4-CDS-F | ATGGCGTTCGTATCTTTCC |
| NbHRLI4-CDS-R | TTAATTTGTTTTTGACTTGGG |
| NbHRLI1-RT-F | TACTAGCCATTGATTCTCTTG |
| NbHRLI1-RT-R | CATGCAGAACTTCAATCACAT |
| NbHRLI2-RT-F | GTTGCTTCTATGATTAACTCT |
| NbHRLI2-RT-R | AATATTCATATTGCACTGCTC |
| NbHRLI6-RT-F | CACTGGTGCTGTTCTACTGAG |
| NbHRLI6-RT-R | GATGTATCCTAAGCTGTCTTG |
| NbHRLI5-RT-F | AGCTTGGCACTCTTTAGTGC |
| NbHRLI5-RT-R | CCTTCTTCTTTGTGGATCG |
| NbHRLI3-RT-F | CGAAGAGAGCAAGCAGTCAG |
| NbHRLI3-RT-R | TGCAATCCAGTGTGTGTCGT |
| NbHRLI4-RT-F | GATTTCAACAGCGCGACAAGA |
| NbHRLI4-RT-R | AAACTTGTGCAGAACTCTAATCAC |
| P3-F | ATGGGAACAGAATGGGAGGA |
| P3-R | TTGATGAACCACCGCCTTTTCT |
| P3C-F | ATGAAGCAATGTATGAAAAG |
| PIPO-F | ATGAAAAAGTTATCTACAA |
| PIPO-R | CTCCGTTCGTAAGATGACATGA |
| P3N-PIPO-F | AGCATCTCCATTTTGGAAAAAAAGTTATC |
| P3N-PIPO-R | GATAACTTTTTTTCCAAAATGGAGATGCT |
| P3N-R | CAAAATGGAGATGCTATGATCC |
