# Supplementary material for: Genome-Wide Identification Reveals That Nicotiana benthamiana Hypersensitive Response (HR)-Like Lesion Inducing Protein 4 (NbHRLI4) Mediates Cell Death and Salicylic Acid-Dependent Defense Responses to Turnip Mosaic Virus
Source: Front Plant Sci. 2021 May 25;12:627315. doi: 10.3389/fpls.2021.627315 (PMC8185164; doi:10.3389/fpls.2021.627315)
Supplement: Supplementary Table 4 — The MEME motif sequence and length of NbHRLIs. [file Table_4.DOC]

Table S4 The MEME Motif Sequence and Length of NbHRLIs

| MOTIF | WIDTH | BEST POSSIBLE MATCH |
| --- | --- | --- |
| MEME-1 | 41 | YQEFSEFGLDGGPAAKALSPKFNVFSKHVATHTGFQVPHVE |
| MEME-2 | 41 | LFFKFTQSLALLGALLFFIGMKNSMPRRSTKKKAPKTKTV |
| MEME-3 | 50 | MKHLILGAJVMKGLGSLLFIFGSSLGAYILLLHQAIASPILYDFYNYDVD |
| MEME-4 | 15 | MAFLSFLGRLLFVSV |
| MEME-5 | 15 | EKEEEEGRERNLKHC |
| MEME-6 | 9 | EKRFKQDPC |
| MEME-7 | 6 | RREQVC |
| MEME-8 | 6 | GRQVPA |
| MEME-9 | 6 | GFQVPK |
| MEME-10 | 6 | GFGGPH |
