# Supplementary material for: Genome-Wide Identification Reveals That Nicotiana benthamiana Hypersensitive Response (HR)-Like Lesion Inducing Protein 4 (NbHRLI4) Mediates Cell Death and Salicylic Acid-Dependent Defense Responses to Turnip Mosaic Virus
Source: Front Plant Sci. 2021 May 25;12:627315. doi: 10.3389/fpls.2021.627315 (PMC8185164; doi:10.3389/fpls.2021.627315)
Supplement: Supplementary Table 7 — Constructs used in Figures 7, 8. [file Table_7.DOCX]

**Table S7** Constructs used in Figure 7 and 8

| Purpose | Constructs combination | remarks |
| --- | --- | --- |
| Y2H | pTSU2-APP+pNubG-Fe65 | Positive control |
|  | pTSU2-APP+pPR3-N | Negative control |
|  | pBT-NbHRLI4+pOst1-NubI | Positive control |
|  | pBT-NbHRLI4+pPR3-N | Negative control |
|  | pBT-NbHRLI4+pPR3-TuMV-P3 |  |
|  | pBT-NbHRLI4+pPR3-TuMV-P3C |  |
|  | pBT-NbHRLI4+pPR3-TuMV-P3N |  |
|  | pBT-NbHRLI4+pPR3-TuMV-P3N-PIPO |  |
|  | pBT-TuMV-P3+pOst1-NubI | Positive control |
|  | pBT-TuMV-P3+pPR3-N | Negative control |
|  | pBT-TuMV-P3+pPR3-NbHRLI4 |  |
|  | pBT-TuMV-P3C+pPR3-NbHRLI4 |  |
|  | pBT-TuMV-P3+pPR3-NbHRLI4^∆7-22^ |  |
|  | pBT-TuMV-P3+pPR3-NbHRLI4^∆63-84^ |  |
|  | pBT-TuMV-P3+pPR3-NbHRLI4^∆88-103^ |  |
|  | pBT-TuMV-P3+pPR3-NbHRLI4^∆120-137^ |  |
| BiFC | pCV-NbHRLI4-YC+pCV-TuMV-P3-YN |  |
|  | pCV-GUS-YCpCV-TuMV-P3-YN | Negative control |
|  | pCV-NbHRLI4-YC+pCV-TuMV-P3C-YN |  |
|  | pCV-GUS-YC+pCV-TuMV-P3C-YN | Negative control |
|  | pCV-NbHRLI4-YC+pCV-GUS-YN | Negative control |
|  | pCV-NbHRLI4-YC+pCV-TuMV-P3N-YN |  |
|  | pCV-NbHRLI4+pCV-TuMV-P3N-PIPO-YN |  |
|  | pCV-NbHRLI4^∆7-22^-YC+pCV-TuMV-P3-YN |  |
|  | pCV-NbHRLI4^∆63-84^-YC+pCV-TuMV-P3-YN |  |
|  | pCV-NbHRLI4^∆88-103^-YC+pCV-TuMV-P3-YN |  |
|  | pCV-NbHRLI4^∆120-137^-YC+pCV-TuMV-P3-YN |  |
| Co-IP | pCV-TuMV-P3-GFP+pCV-NbHRLI4-flag |  |
|  | pCV-TuMV-P3C-GFP+pCV-NbHRLI4-flag |  |
|  | pCV-TuMV-P3N-GFP+pCV-NbHRLI4-flag |  |
|  | pCV-TuMV-P3N-PIPO-GFP+pCV-NbHRLI4-flag |  |
|  | pCV-GUS-GFP+pCV-NbHRLI4-flag | Negative control |
